# Gestational diabetes mellitus in relation to serum per- and polyfluoroalkyl substances: A scoping review to evaluate the need for a new systematic review

**DOI:** 10.12688/f1000research.144376.2

**Published:** 2024-10-09

**Authors:** Ghazaleh Aali, Anna K. Porter, Sebastian Hoffmann, Matthew P. Longnecker, Farhad Shokraneh

**Affiliations:** 1 Department of Evidence Synthesis, Systematic Review Consultants LTD, Oxford, UK; 2 Ramboll U.S. Consulting, Inc., Raleigh, NC, USA; 3 seh consulting + services, Paderborn, Germany

**Keywords:** Perfluoroalkyl, Polyfluoroalkyl, Gestational Diabetes Mellitus, Glucose, Environmental Epidemiology, Exposure

## Abstract

**Background:**

Per- and polyfluoroalkyl substances (PFAS) were used or are being used in the manufacturing of products, including consumer-use products. The resulting environmental contamination has led to widespread human exposure. This review aimed to scope the characteristics of evidence covered and applied methodology of evidence to understand -- regardless of any results on the association of gestational diabetes mellitus (GDM) and PFAS -- if a new systematic review would be justified.

**Methods:**

We systematically identified reports investigating associations of PFAS with GDM following a pre-specified and pre-registered PECO framework and protocol.

**Results:**

The previous systematic reviews included 8-11 reports and either conducted meta-analyses stratified by comparator, analyzed results based on only high and low exposure categories, or pooled results across comparators. Included 20 reports presented data on 24 PFAS with PFOA, PFOS, PFHxS, PFNA, and PFDA being examined in almost all. The comparators employed were heterogeneous across the reports.

**Conclusions:**

Because data from at least one new report on GDM is available since the previous systematic reviews and heterogeneous comparators, an updated systematic review using SWiM could add value to the literature.

## Introduction

Per- and polyfluoroalkyl substances (PFAS) were used or have ongoing use in the manufacturing of a variety of products, including consumer-use products. ^1^^,^^2^ Almost everyone in developed countries has a detectable amount of PFAS in their blood serum due to exposure via contaminated food and other sources, and the unreactivity of these chemicals. ^3^ Despite some being phased out of production, ^4^ PFAS are widespread and have been detected in different continents irrespective of the level of industrialization, indicating long-range atmospheric transport as an important pathway of PFAS distribution. ^5^

Serum or plasma concentrations of PFAS have been associated with several health outcomes, including gestational diabetes mellitus (GDM). ^6^^–^^11^ GDM increases risk among offspring of preterm birth and macrosomia, ^12^ and, among mothers, of type 2 diabetes mellitus. ^13^ In the U.S., the proportion of pregnant women who develop GDM has increased over the past ten years, possibly due to trends in risk factors such as obesity, but environmental factors could be contributing. ^14^

Our recent experience in evaluating systematic reviews of the relation of health outcomes to PFAS serum concentrations has made us aware of the variety of comparators used in original reports. ^15^ This variety poses challenges in summarizing data that have not been adequately addressed in the GDM-PFAS systematic review literature. Thus, in addition to identifying relevant original reports in this scoping review, we addressed our special interest in the methods used in previous meta-analyses – but not in their findings.

### Motivation and rationale

Funding for this scoping review was provided by 3M. 3M previously used perfluorooctyl and perfluorohexyl chemistries in manufacturing and has more recently used precursors of PFBS. 3M has publicly communicated that they will exit all PFAS manufacturing by the end of 2025. ^16^ 3M would like to gain insights as to the strengths and weaknesses of the collective epidemiologic research on gestational diabetes mellitus in relation to PFAS. 3M requested a highly rigorous scoping review to better understand the nature of the existing data and evaluate the need for a new systematic review. The sponsor will not be involved in the conduct of the work at any stage and will not see our work products until they are published in a peer-reviewed journal. Ramboll provides updates regarding progress toward publication and required resources.

If PFAS are a risk factor for GDM, this might affect regulations on allowable amounts of PFAS in water or food. GDM was not considered a critical endpoint in a recent U.S. government evaluation, but a more recent European government evaluation indicated that it could be. ^4^^,^^17^ Our objective was to collect and summarize the research on the relationship between serum per- and polyfluoroalkyl substances and gestational diabetes mellitus with the aim to inform the necessity of systematically reviewing the topic, potentially including a meta-analysis, and to estimate the required investment of resources needed for such a review.

## Methods

We followed Joanna Briggs Institute’s guidance for designing and conducting this scoping review ^18^ and followed PRISMA-P, ^19^ PRISMA-ScR, ^20^ PRISMA-Abstract, ^21^ and PRISMA-Search ^22^ in reporting the protocol and the final report of this review. The indications for a scoping review were: a) to examine how research is conducted on a certain topic or field, and b) as a precursor to a systematic review. ^23^

We used the PECO framework (Population, Exposure, Comparator, and Outcomes) ^24^ for formulating the problem to be addressed. The PECO also guided setting the eligibility criteria ( Table 1). The population used was pregnant women. The exposure was the concentration of any specific PFAS in serum or plasma during pregnancy. The measure of exposure was obtained from a blood specimen or, for populations in which a validated pharmacokinetic model of serum concentration was available, estimated on an individual basis. Estimated serum or plasma PFAS for an individual from a population for which a validated pharmacokinetic model of serum concentration was available is acceptable because in the absence of a serum or plasma measurement, pharmacokinetic models can be useful for imputation when a population water supply had unusual high PFAS concentrations. ^25^ The comparator was a contrast in serum PFAS concentration that was either per arithmetic unit of PFAS (e.g., ng/ml), per log unit increase, or categorical. The primary outcome was GDM, and the secondary outcome was serum glucose.

**Table 1.** Eligibility and exclusion criteria of this scoping review according to the PECO framework.

	Eligibility criteria	Exclusion criteria ^a^
Population	Pregnant women regardless of age, health status, ethnicity, occupation, and socioeconomic status	Any study not including pregnant women
Exposure	Concentrations of any specific PFAS in serum or plasma during pregnancy; measured or estimated PFAS may include PFAS measured outside of pregnancy in consideration of the long half-life of many PFAS	Externally measured or estimated PFAS exposure concentrations, such as food, water, or air that are not translated to PFAS serum or plasma concentrations for specific people; mixture analyses
Comparator	An incremental increase in concentration of a specific PFAS compared with a lower concentration, across the entire range of exposure; systematic reviews with high:low exposure comparators will be included	None ^b^
Outcomes		
• Gestational Diabetes Mellitus (primary)	Gestational Diabetes Mellitus as defined by original authors	None ^b^
• Serum glucose (secondary)	Measured glucose	None ^b^
Study type	Case-control, cross-sectional, or cohort designs	Ecologic studies, case studies, reviews, laboratory reports, letters

^a^ See Methods for an elaboration on the exclusion criteria.

^b^ Other than those implied by the eligibility criteria.

Observational studies such as case-control, longitudinal (cohort, prospective, retrospective), or cross-sectional studies were included in this review. Full reports about original epidemiologic research or meta-analyses had to be published online or in print. Pre-clinical studies (cells or genetic material) and studies with non-human subjects were excluded. If the data were for a non-pregnant population only or a mixed population where separate data on the pregnant population was unavailable, we excluded the report. If a measured or estimated serum or plasma concentration of PFAS in the person was not examined in relation to the occurrence of GDM or blood glucose in the person the report was excluded. Mixture analyses where there were no single exposure analyses for PFAS reported in that paper were excluded. ^26^ We excluded narrative and systematic narrative reviews; however, before the exclusion, we checked for any relevant unique included reports in the systematic narrative reviews. To identify relevant ongoing or published systematic reviews to examine their methods, we conducted a rapid search in PROSPERO and PubMed.

On 11 ^th^ December 2022, we systematically searched the latest available version of the following sources to identify original reports without date, time, document type, or language limitations ^27^: CINAHL via EBSCOhost (1937 – search date); Embase via Ovid SP (1974 – 9 ^th^ December 2022); Emerging Sources Citation Index via Web of Science (2015 – search date); MEDLINE via Ovid SP (1946 – 9 ^th^ December 2022); PubMed (excluding MEDLINE; 1946 – search date); and Science Citation Index Expanded via Web of Science (1900 – search date).

An information scientist designed the search strategies using the terms collected from the experts, literature, and controlled vocabularies (CINAHL Headings, Medical Subject Headings=MeSH, and Emtree). The search strategy for MEDLINE was shared with two experts on the topic for commenting and revisions and peer-reviewed by another information specialist based on PRESS guidance ^28^ before being translated into the syntax of other databases.

The search strategies (See the *Extended data*) were tested and tuned based on a randomly selected half of the studies included in the previous systematic reviews. The final search was tested against the remaining half of the included studies from previous reviews to ensure the comprehensiveness of the retrieval.

Results were imported to the reference management software, EndNote X9, for de-duplication. The de-duplicated search results were imported into Rayyan ^29^ and two reviewers screened the search results independently after turning the blinding mode on. We followed the same procedure for screening the full reports of the studies. For any disagreement, the two reviewers discussed and agreed on a final decision to include or exclude the report.

Data extraction was designed, piloted, and revised to include the following data points: study name (first author’s last name and publication year), study design, study period, country of origin, participants’ age, number of participants per group, duration of the pregnancy at the baseline of the study, time of glucose measure, name of the glucose test, diagnostic criteria for GDM, sample type for exposure measurement, time of measuring PFAS, ethnicity, setting (rural, urban), source of exposure, length of exposure, name of substances, summary statistic for PFAS and glucose concentrations per group, exposure data type, chemical analysis methods, measures of association, exposure metric (comparator), potentially relevant references for identifying more relevant studies, the lead author’s name and email address, and any relevant notes.

When we could not find the required data in the report, we contacted the authors to request it.

We summarized the findings narratively or in tables. Because this was a scoping review, we did not run any data analysis or risk of bias appraisal for the included studies.

## Results

We identified three previously published systematic reviews that explored the GDM-PFAS association ( Table 2). ^30^^–^^32^ In one systematic review (Yan *et al.*, 2022), ^32^ the use of meta-analysis to combine results across comparators rendered the meaning of the summary OR obscure. In another systematic review (Gao *et al.*, 2021), ^30^ results from different evidence streams were not combined using an appropriate summary. In the third systematic review (Wang *et al.*, 2022), the focus on only high:low exposure category comparators meant: a) informative data from each included study were ignored, b) the variability of the exposure contrasts made use of this comparator questionable, and c) reports using only models with exposure as a continuous variable were ignored. The most recent of the systematic reviews had a literature search end date of November 2021. None of the previous systematic reviews employed synthesis without meta-analysis (SWiM). ^33^ Although our focus was not on the results of the previous systematic reviews, a summary of their results was as follows: a) no significant association was found between PFAS and GDM ^30^; b) PFOA was significantly associated with higher risk of GDM ^31^; and c) PFOA and PFBS increase risk of GDM. ^32^

**Table 2.** Characteristics of previous systematic reviews of GDM and PFAS.

1 ^st^ author, year	N of studies ^a^	Literature review end date	Results combined across comparators ^b^	Results stratified by comparator	Results based on single comparator	Used synthesis without meta-analysis
Gao 2021	9	Feb 21	N	Y	N	N
Wang 2022	8	Sep 20	N	N	Y ^c^	N
Yan 2022	11	Nov 21	Y	N	N	N

^a^ All eight studies included in Wang *et al*. (2022) are in Gao *et al*. (2021), and all nine studies included in Gao *et al*. (2021) are included in Yan *et al*. (2022).

^b^ Meta-analysis done across results based on different types of comparators.

^c^ Wang *et al*. (2022) conducted a meta-analysis based on a high:low exposure category comparator.

Four protocols registered in PROSPERO either explicitly addressed GDM and PFAS or might have included it in a broader definition of outcome or exposure ( Table 3). Two of the protocols were old enough that it was likely the effort had been abandoned; of the more recent protocols, one did not specifically mention the inclusion of pregnant women or gestational diabetes. The remaining study described the use of a high:low exposure category comparator, which is statistically inefficient because it does not include all available results from a report and has other limitations (see Discussion). None of the studies mentioned use of SWiM. ^33^

**Table 3.** Protocols for systematic reviews registered in PROSPERO (updated on 14 ^th^ June 2023).

Reg. Number	Title	End date	Use of SWiM	Corresponding publication *	Notes
CRD42020192570	Investigating the relationship between maternal exposure to persistent organic pollutants (POPs) during pregnancy and gestational diabetes	3-30-21	Not mentioned	No	
CRD42021233141	Association of Per-polyfluoroalkyl substances with gestational diabetes mellitus and diabetes-related metabolic traits in pregnant women: a systematic review and meta-analysis	5-30-21	Not mentioned	No	
CRD42022332561	Association of Perpolyfluoroalkyl exposure with diabetes mellitus prevalence and its related complications: a systematic review and meta-analysis [sic]	9-30-23	Not mentioned	No	No mention of pregnancy or gestation in protocol
CRD42022369711	Exposure to Per- and Polyfluoroalkyl Substances (PFAS) and Diabetes Mellitus: A Systematic Review and Meta-Analysis	1-1-23	Not mentioned	No	Will use high:low exposure categories as comparator

* Per PubMed search 6-16-23.

**Figure 1.**
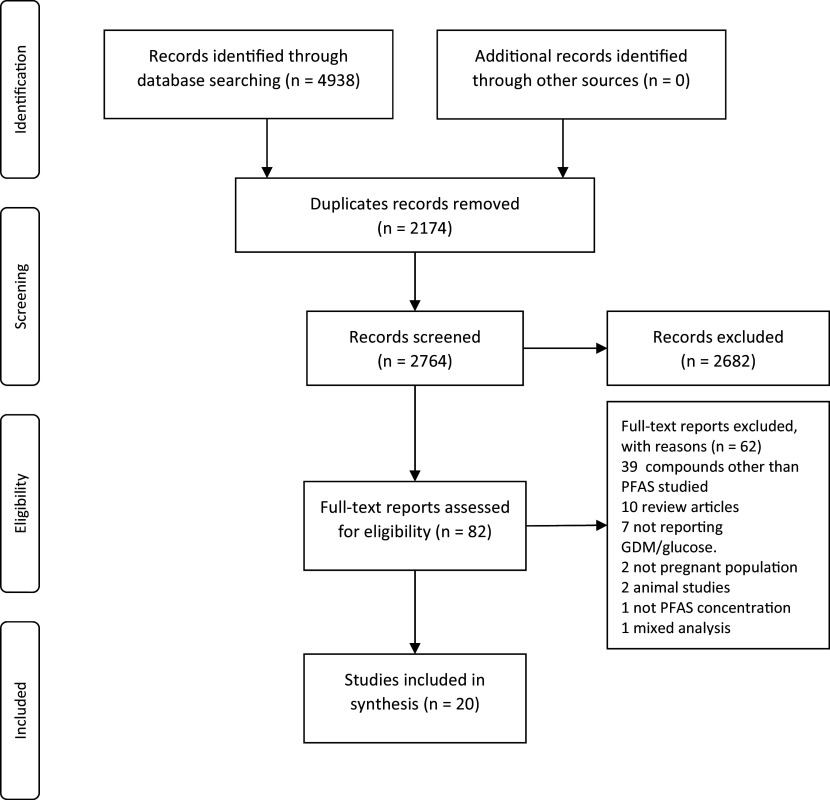
The PRISMA diagram for the process of selecting the studies.

The search for original data reports found 4938 records. After removing the duplicates, we screened the unique records and identified 82 possibly relevant records based on their titles and abstracts. When the full-text reports were obtained for these 82 records, we excluded 62 after documenting the reason for exclusion and included 20 reports in this review. ^6^^–^^11^^,^^34^^–^^47^ The process of selection of reports is shown in Figure 1.

Two reports used the data from the Shanghai Birth Cohort ^11^^,^^45^ with the possibility of duplicate and overlapping data. The source and length of exposure were not reported in any of the reports, presumably indicating that all were of populations with background exposure.

Characteristics of the reports are presented in Table 4. Based on these data, the prospective cohort (12 reports) was the most used study design followed by case-control (5). The studies were conducted between 1997 and 2021 in China (10 reports), North America (US (6) & Canada (1)), Denmark (2), and Spain (1). The 10 studies from China all had Chinese participants, the ethnicity of participants from all seven North American studies was diverse, one of the studies from Denmark included only participants of Danish ethnicity, while the other did not report participant ethnicity, and the one study from Spain had participants of primarily Spanish origin.

**Table 4.** Characteristics of included reports.

Study	Design	Period	Country	Age	Participants	Controls	Pregnancy duration ^a^	Ethnicity	Setting
**Zhang 2015**	Prospective Cohort (With Control Group)	2005-2009	US	29.7 ± 3.7	258 pregnant women (28 GDM)	230 Non-GDM	NA ^b^	White, Non-White	NA
**Shapiro 2016**	Prospective Cohort (With Control Group)	2008-2011	Canada	33.0 ± 4.9	1274 pregnant women (59 GDM)	1167 Normal Glucose	6 to < 14 GW	White, Non-White	Urban
**Matilla-Santander 2017**	Prospective Cohort (No Control Group)	2003-2008	Spain	31.9 ± 4	1240 pregnant women (53 GDM)	NA	1st Trimester	Spanish	Urban/mixed
**Starling 2017**	Prospective Cohort (No Control Group)	2009-2014	US	27.8 ± 6:2 Age at Delivery	652 pregnant women (628 with fasting glucose)	NA	<24 GW	Black/Non-Hispanic, Hispanic, White/Non-Hispanic, Others	Urban/mixed
**Valvi 2017**	Cohort (Retrospective Data, With Control Group)	1997-2000	Denmark	29.2 ± 5.2 Age at Delivery	604 pregnant women (49 GDM)	555 Non-GDM	34 GW	Faroese	Mixed
**Jensen 2018**	Prospective Cohort (With Control Group)	2011 (Sep) - 2013 (Sep)	Denmark	29.9 ± 4.4	158 pregnant women with high GDM risk (all with OGTT ^c^)	160 Low GDM Risk	8-16 GW	Danish	Urban/mixed
**Wang 2018a**	Prospective Cohort (Repeat Measurement-Based; No Control Group)	2013 (Sep) - 2014 (Dec)	China	20-40	560 pregnant women (35 GDM)	NA	5-15 GW	Chinese	Urban
**Wang 2018b**	Case-Control	2013 (Jan) - 2013 (Mar)	China	29.5 (28.0 - 32.0) GDM 29.0 (28.0-31.0) Non-GDM	252 pregnant women (84 GDM)	168 Healthy	NA	Chinese	Urban
**Liu 2019**	Case-Control (Nested)	2013 (Aug) - 2015 (Jun)	China	29.3 ± 2.9	439 pregnant women (63 GDM)	126 Healthy	1st Trimester	Chinese	Urban
**Rahman 2019**	Prospective Cohort (No Control Group)	2009 (Jul) - 2013 (Jan)	US	28.2 ± 5.5	2334 pregnant women (74 GDM)	NA	8-13 GW	Asian, Black/Non-Hispanic, Hispanic, White/Non-Hispanic	NA
**Li 2020**	Prospective Cohort (No Control Group)	2013 (Oct) - 2014 (Aug)	China	28.3 ± 3.2	874 pregnant women (59 GDM)	NA	24-28 GW	Chinese	Urban
**Preston 2020b**	Prospective Cohort (No Control Group)	1999-2002	US	31.9 ± 5.1	1540 pregnant women (85 GDM)	NA	22 ≥ GW	Asian, Black, Hispanic, White, Other	Urban
**Ren 2020**	Cohort (Retrospective Data, No Control Group)	2012 (Apr) - 2012 (Dec)	China	27.8 ± 3.3	981 pregnant women (PFAS); 856 (FG); 705 (1hG)	NA	12-16 GW	Chinese	Urban
**Xu 2020**	Case-Control (Nested in Prospective Cohort)	2017 (Jul 1) - 2019 (Jan 31)	China	29.7 ± 3.1	2460 pregnant women (165 GDM)	330 Healthy	24-28 GW	Chinese	Urban
**Mehta 2021**	Prospective Cohort (No Control Group)	2011-2015	US	≤27	95 overweight and obese pregnant women (all with fasting glucose)	NA	<14 GW	Black/Non-Hispanic, Latina, White/Non-Hispanic	Urban
**Vuong 2021**	Cohort (Retrospective Data, No Control Group)	2003 (Mar) - 2006 (Feb)	US	<25: 93 25-34: 229 ≥35: 61	388 pregnant women (234 with non-fasting glucose)	NA	16 ± 3 GW	Black/Non-Hispanic, White/Non-Hispanic, Others	Urban
**Yu 2021**	Prospective Cohort (With Control Group)	2013-2016	China	29.1 ± 3.7	2747 pregnant women (325 GDM)	2422 Non-GDM	15 (13-17) GW Median (IQR)	Chinese	Urban
**Xu 2022**	Case-Control	2020 (Oct) - 2021 (Sep)	China	31.5 ± 4.38	340 pregnant women (171 GDM)	169 Healthy	271 ± 7.22 (Days)	Chinese	Urban
**Wang 2023a**	Prospective Cohort (No Control Group)	2013-2016	China	29.4 ± 3.8	1405 pregnant women (270 GDM)	NA	39.1 ± 1.4 GW	Chinese	Urban
**Zhang 2023**	Case-Control	2011 (Jul) - 2012 (Nov)	China	34.4 ± 4.6 (GDM) 30.9 ± 4.6 (Non-GDM)	204 pregnant women (135 GDM)	69 Healthy	38.3 ± 1.6 GW (GDM) 38.2 ± 1.8 GW (Non-GDM)	Chinese	Urban

^a^ At study baseline.

^b^ NA: Not available.

^c^ OGTT: Oral Glucose Tolerance Test.

The 20 reports included a total of 18,805 participants. Among the 15 reports that presented the number of participants with GDM, the proportion with GDM was 10% (1,655/16,513); the other five reports presented results on serum glucose only and not GDM. The number of participants across the reports ranged between 95 and 2747. The participants’ age was between 20 and 40 with the most frequent mean age reported being between 28 and 32.

Participants’ duration of pregnancy at study baseline varied between 5 and 38 weeks making it one of the most heterogenous items of data extracted.

Table 5 summarizes the list of 24 PFAS (not counting isomers) for which a measure of association was reported in the included studies with results for PFOA, PFOS, PFHxS, PFNA, and PFDA being reported in almost all the studies.

**Table 5.** PFAS in included reports for which a measure of association with an outcome was presented.

Acronyms for Specific PFAS ^a^	CAS Number ^b^	No. Carbons ^c^	Jensen 2018	Li 2020	Liu 2019	Matilla-Santander 2017	Mehta 2021	Preston 2020b	Rahman 2019	Ren 2020	Shapiro 2016	Starling 2017	Valvi 2017	Vuong 2021	Wang 2018a	Wang 2018b	Wang 2023a	Xu 2020	Xu 2022	Yu 2021	Zhang 2015	Zhang 2023	Total
**Carboxylic Acids**																							
**PFBA** ^d^	375-22-4	4	-	-	-	-	-	-	-	-	-	-	-	-	-	-	-	-	-	-	-	✓	1
**PFPeA** ^e^	2706-90-3	5	-	-	-	-	-	-	-	-	-	-	-	-	-	-	-	-	-	-	-	✓	1
**PFHxA** ^f^	307-24-4	6	-	-	-	-	-	-	-	-	-	-	-	-	-	-	-	-	-	-	-	✓	1
**PFHpA** ^g^	375-85-9	7	-	✓	-	-	-	-	✓	-	-	-	-	-	-	-	-	-	✓	✓	-	✓	5
**ADONA** ^h^	919005-14-4	7	-	-	-	-	-	-	-	-	-	-	-	-	-	-	-	-	✓	-	-	-	1
**PFOA (sum)** ^i^	355-67-1	8	✓	✓	-	✓	✓	✓	✓	✓	✓	✓	✓	✓	✓	-	✓	✓	✓	✓	✓	✓	18
** n-PFOA** ^j^	355-67-1		-	-	✓	-	-	-	-	-	-	-	-	-	-	✓	-	-	-	-	-	-	2
** 6m-PFOA** ^k^	15166-06-0		-	-	✓	-	-	-	-	-	-	-	-	-	-	-	-	-	-	-	-	-	1
**PFNA** ^l^	375-95-1	9	✓	✓	-	✓	✓	✓	✓	✓	-	✓	✓	✓	-	✓	✓	✓	✓	✓	✓	✓	17
**PFDA (PFDeA)** ^m^	335-76-2	10	✓	✓	-	-	✓	-	✓	✓	-	✓	✓	-	-	✓	✓	✓	✓	✓	✓	✓	14
**PFUnDA** ^n^	2058-94-8	11	-	✓	-	-	-	-	✓	✓	-	-	-	-	-	✓	✓	✓	✓	✓	-	-	8
**PFDoA (PFDoDA)** ^o^	307-55-1	12	-	✓	-	-	-	-	✓	✓	-	-	-	-	-	-	-	✓	✓	✓	-	✓	7
**PFTrDA (PFTriDA)** ^p^	72629-94-8	13	-	✓	-	-	-	-	-	✓	-	-	-	-	-	-	-	-	✓	-	-	✓	4
**PFTeDA** ^q^	376-06-7	14	-	✓	-	-	-	-	-	-	-	-	-	-	-	-	-	-	-	-	-	✓	2
**Sulfonic Acids**																							
**PFBS** ^r^	375-73-5	4	-	✓	-	-	-	-	-	-	-	-	-	-	-	-	-	✓	-	✓	-	✓	4
**PFHxS** ^s^	355-46-4	6	✓	✓	-	✓	✓	✓	✓	✓	✓	✓	✓	✓	-	✓	✓	✓	✓	✓	-	✓	17
**4:2FTS** ^t^	757124-72-4	6	-	-	-	-	-	-	-	-	-	-	-	-	-	-	-	-	✓	-	-	-	1
**PFHpS** ^u^	375-92-8	7	-	-	-	-	-	-	-	-	-	-	-	-	-	-	-	✓	-	-	-	-	1
**PFOS (sum)** ^v^	1763-23-1	8	✓	✓	-	✓	✓	✓	✓	✓	✓	✓	✓	✓	✓	-	✓	✓	✓	✓	✓	✓	18
** n-PFOS** ^w^	1763-23-1		-	-	✓	-	-	-	-	-	-	-	-	-	-	✓	-	-	-	-	-	-	2
** 1m-PFOS** ^x^	1763-23-1		-	-	-	-	-	-	-	-	-	-	-	-	-	✓	-	-	-	-	-	-	1
** 3m+4m-PFOSc** ^aa^	1763-23-1		-	-	-	-	-	-	-	-	-	-	-	-	-	✓	-	-	-	-	-	-	1
** 5m-PFOS** ^bb^	1763-23-1		-	-	-	-	-	-	-	-	-	-	-	-	-	✓	-	-	-	-	-	-	1
** 6m-PFOS** ^cc^	1763-23-1		-	-	-	-	-	-	-	-	-	-	-	-	-	✓	-	-	-	-	-	-	1
**6:2 Cl-PFESA** ^dd^	73606-19-6	8	-	✓	-	-	-	-	-	-	-	-	-	-	-	-	-	-	✓	-	-	✓	3
**6:2FTS** ^ee^	27619-97-2	8	-	-	-	-	-	-	-	-	-	-	-	-	-	-	-	-	✓	-	-	-	1
**8:2 Cl-PFESA** ^ff^	83329-89-9	10	-	✓	-	-	-	-	-	-	-	-	-	-	-	-	-	-	-	-	-	✓	2
**PFDS** ^gg^	335-77-3	10	-	-	-	-	-	-	-	-	-	-	-	-	-	-	-	-	✓	-	-	-	1
**Sulfonamides and Sulfonamidoacedic Acids**																							
**PFOSA** ^hh^	754-91-6	8	-	-	-	-	-	-	-	-	-	-	-	-	-	-	-	-	-	-	✓	✓	2
**MeFOSAA (Me-PFOSA-AcOH)** ^ii^	2355-31-9	11	-	-	-	-	-	✓	✓	-	-	-	-	-	-	-	-	-	-	-	✓	-	3
**EtFOSAA (Et-PFOSA-AcOH)** ^jj^	2991-50-6	12	-	-	-	-	-	✓	-	-	-	-	-	-	-	-	-	-	-	-	✓	-	2

^a^ Frequently used alternative acronyms are in parentheses.

^b^ For PFOA and PFOS, isomer-specific CAS#s were not available at this time this manuscript was prepared (except for 6m-PFOS); we followed CDC’s example of using the same CAS# for the isomers of PFOA (sum) and PFOS (sum) ( https://www.cdc.gov/nceh/dls/oatb_capacity_14.html).

^c^ Number of carbons not given for isomers.

^d^ PFBA (PFBA (C4)): Perfluorobutanoic Acid.

^e^ PFPeA (PFPeA (C5)): Perfluoropentanoic Acid.

^f^ PFHxA (PFHxA (C6)): Perfluorohexanoic Acid.

^g^ PFHpA (PFHpA (C7)): Perfluoroheptanoic Acid.

^h^ ADONA: 4,8-Dioxa-3H-Perfluorononanoic Acid.

^i^ PFOA (PFOA (C8)): Perfluorooctanoic Acid.

^j^ n-PFOA: N-Perfluorooctanoic Acid.

^k^ 6m-PFOA: Perfluoro-6-Methylpheptanoic Acid.

^l^ PFNA (PFNA (C9)): Perfluorononanoic Acid.

^m^ PFDA (PFDeA or PFDA (C10)): Perfluorodecanoic Acid.

^n^ PFUnDA (PFUnDA (C11)): Perfluoroundecanoate.

^o^ PFDoA (PFDoDA or PFDoDA (C12)): Perfluorododecanoic Acid.

^p^ PFTrDA (PFTriDA (C13) or PFTrDA (C13)): Perfluorotridecanoate.

^q^ PFTeDA (C14): Perflurotetradecanoate.

^r^ PFBS (PFBS (C4)): Perfluorobutanesulfonic Acid.

^s^ PFHxS (PFHxS (C6)): Perfluorohexanesulfonic Acid.

^t^ 4:2FTS: 4:2 Fluorotelomer Sulfonic Acid.

^u^ PFHpS: Perfluoroheptane Sulfonic Acid.

^v^ PFOS (PFOS (C8)): Perfluorooctanesulfonic Acid.

^w^ n-PFOS: N-Perfluorooctane Sulfonate.

^x^ 1m-PFOS: Perfluoro-1-Methylheptanesulfonate.

^y^ 3m-PFOS: Perfluoro-3-Methylheptanesulfonate.

^z^ 4m-PFOS: Perfluoro-4-Methylheptanesulfonate.

^aa^ 3m+4m-PFOSc: Perfluoro-3-Methylheptanesulfonate + Perfluoro-4-Methylheptanesulfonate.

^bb^ 5m-PFOS: Perfluoro-5-Methylheptanesulfonate.

^cc^ 6m-PFOS: Perfluoro-6-Methylheptanesulfonate.

^dd^ 6:2 Cl-PFESA: 6:2 Chlorinated Polyfluorinated Ether Sulfonate.

^ee^ 6:2FTS: 6:2 Fluorotelomer Sulfonic Acid.

^ff^ 8:2 Cl-PFESA: 8:2 Chlorinated Polyfluorinated Ether Sulfonic Acid.

^gg^ PFDS: Perfluorodecane Sulfonate.

^hh^ PFOSA: Perfluorooctanesulfonamide.

^ii^ MeFOSAA (Me-PFOSA-AcOH): Methylperfluorooctanesulfonamidoacetic Acid.

^jj^ EtFOSAA (Et-PFOSA-AcOH): Ethylperfluorooctane Sulfonamidoacetic Acid.

Table 6 presents the data regarding the measurement and reporting of PFAS concentration, glucose, and GDM. An oral glucose tolerance test (OGTT) was used in 16 reports as the glucose test and seven reports followed the International Association of Diabetes and Pregnancy Study Groups (IADPSG) criteria for diagnosing GDM. Among 12 reports that reported type of glucose measure, 10 reported fasting glucose (FG), six reported one hour glucose (1hG), and five reported two hour glucose (2hG), most of which reported mean ± standard deviation (nine reports). While the time for measuring glucose levels varied between 14 weeks of gestation and 1-2 days pre-delivery, measurement of PFAS was in a wider time range from 8 weeks of gestation to delivery. HPLC LC-MS/MS was the most-used chemical analysis method for PFAS. A summary statistic for PFAS concentrations was presented for 19 reports with the geometric mean being the most frequently reported (10) followed by the median (seven reports). Standard deviation was presented in only four reports with geometric means and interquartile range (IQR) for six reports with median.

**Table 6.** Measures of glucose and PFAS in the included reports.

Study	Glucose measure time	Glucose test	GDM Diagnostic criteria	Chemical measure Time	PFAS statistic	Exposure categories	Chemical analysis method	Glucose measures	Glucose statistic	Measures of association	Exposure metric (Comparator)
**Zhang 2015**	NA	NA	Self-Report (Physician Diagnosis)	NA	Geometric Mean (95% CI)	NA	HPLC LC-MS/MS	NA	NA	Crude and adjusted OR (GDM)	Per SD ln-unit difference in PFAS concentration
**Shapiro 2016**	NA	OGTT	Canadian Diabetes Association and the Society of Obstetricians and Gynaecologists of Canada	1st Trimester	Geometric Mean (SD)	Quartiles	UPLC/MS-MS	NA	NA	Crude and adjusted OR (IGT and GDM)	By quartile of PFAS concentration
**Matilla-Santander 2017**	24-28 GW	OGTT	Spanish Group of Diabetes and Pregnancy	13 GW (13:1 ± 1:4)	Geometric Mean (SD)	Quartiles	HPLC LC-MS/MS	NA	NA	Minimally adjusted ^a^ and fully adjusted OR (IGT and GDM)	By quartile & per log10-unit difference in PFAS concentration
**Starling 2017**	27 (20-34) GW Median (Range)	NA	NA	27 (20-34) GW Median (Range)	Geometric Mean (95% CI)	Tertiles	Online Solid Phase Extraction-PLC-Isotope Dilution-Tandem MS (Modified)	FG	Mean ± SD	Crude and adjusted β (FG)	By terile & per ln-unit difference in PFAS concentration
**Valvi 2017**	24-28 GW	OGTT	SOGC Clinical Practice Guidelines on Screening for Gestational Diabetes Mellitus	34 GW	Median (IQR)	Tertiles	LC with Tandem MS	NA	NA	Crude and adjusted OR (GDM)	By tertile & per log2-unit difference in PFAS concentration
**Jensen 2018**	28 GW	OGTT	European Association for the Study of Diabetes (Lind and Phillips, 1991)	11.3 (9.9; 14.3) GW (Median IQR)	Median (5th - 95th Percentile)	NA	Online Solid Phase Extraction followed by LC-MS/MS	FG 1hG 2hG	Median (IQR)	Crude and adjusted percent difference (FG, 1hG, 2hG)	Per log2-unit difference in PFAS concentration
**Wang 2018a**	21-26 GW; 26.8 ± 2.2 (26) Mean ± SD (Median)	OGTT (n=385)	IADPSG	Middle Term of Pregnancy	Range	Tertiles	UPLC-Q/TOF MS	FG (Average) OGTT (Average)	Mean ± SD	Crude and adjusted β (FG, OGTT) and HR (GDM)	By terile (GDM) & per log-unit difference in PFAS concentration (FG, OGTT)
**Wang 2018b**	1-2 Days Pre-Delivery	OGTT	Ministry of Health China Criteria (WS311-2011), based on IADPSG	1-2 Days Pre-Delivery	Median (IQR)	Dichotomized	NA	NA	NA	Crude and adjusted OR (FG and GDM)	Dichotomized (FG) & per 1 ng/mL difference in PFAS concentration (GDM)
**Liu 2019**	24-28 GW	OGTT	Ministry of Health China Criteria (WS311-2011), based on IADPSG	1st Trimester	Median (IQR)	Tertiles	HPLC LC-MS/MS	FG 1hG 2hG	Mean ± SD	Conditioned ^b^ and adjusted OR (GDM) and adjusted β (FG, 1hG, 2hG)	By tertile (GDM) & per ln-unit difference in PFAS concentration (GDM, FG, 1hG, 2hG)
**Rahman 2019**	27.5 ± 4.3 GW	OGTT	Carpenter and Coustan	8-13 GW	Geometric Mean (95% CI)	NA	LC-SPE-LC-MS/MS	NA	NA	Adjusted RR (GDM)	Per 1 SD-unit difference in PFAS concentration
**Li 2020**	24-28 GW	OGTT	IADPSG	Delivery	Geometric Mean	Dichotomized	HPLC	FG 1hOGTT 2hOGTT	NA	Crude and adjusted β (FG, 1hG, 2hG) and OR (GDM)	Per log2-unit difference in PFAS concentration
**Preston 2020b**	28 GW	OGTT	Carpenter and Coustan	9.7 GW (Median)	NA	Quartiles	NA	GCT Blood Glucose	Mean ± SD	Adjusted OR (IGT and GDM) and β (1hG)	By quartile (IGT, GDM, 1hG) & per ln-unit difference in PFAS concentration (IGT, GDM)
**Ren 2020**	12-20 GW (FG); 20-28 GW (1hG)	OGTT	Medical Records at Birth	12-16 GW	Geometric Mean (SD)	Tertiles	HPLC LC-MS/MS	FG 1hG	Mean ± SD	Crude and adjusted OR (FG and 1hG)	Per ln-unit difference in PFAS concentration
**Xu 2020**	24-28 GW	OGTT	IADPSG	16-20 GW	Median	Quartiles	UPLC-Q/TOF MS	NA	NA	Crude and adjusted OR (GDM)	By quartile & per log10-unit difference in PFAS concentration
**Mehta 2021**	<14 GW	OGTT	NA	16 (10-24) GW Median (Range)	Geometric Mean (SE)	NA	LC-SPE-LC-MS/MS	FG	Geometric Mean (SE)	Adjusted percent difference (FG)	Per log2-unit difference in PFAS concentration
**Vuong 2021**	≥ 26 GW (n=234)	OGTT	NA	16 or 26 GW	Geometric Mean (SD)	NA	HPLC-Isotope Dilution-Tandem MS	Glucose at ≥ 26	Mean ± SD	Adjusted β (1hG)	Per ln-unit difference in PFAS concentration
**Yu 2021**	24-28 GW	NA	IADPSG	15 (13-17) GW Median (IQR)	Median (IQR)	Tertiles	HPLC LC-MS/MS	FG 1hG 2hG	Mean ± SD	Adjusted OR (GDM) Adjusted β (FG, 1hG and 2hG)	Per ln-unit difference in PFAS concentration
**Xu 2022**	271 ± 7.22 (Days)	OGTT	IADPSG	1-2 Days Pre-Delivery	Median (IQR)	Tertiles	UPLC/MS-MS	FG 1hG 2hG	Mean ± SD	Crude and adjusted OR (GDM) Adjusted β (FG, 1hG and 2hG)	By tertile of PFAS concentration
**Wang 2023a**	24-28 GW	NA	IADPSG	15 (9-16) GW Median (Range)	Geometric Mean (Range)	NA	HPLC LC-MS/MS	FG	Mean ± SD	Crude and adjusted β (FG)	Per log2-unit difference in PFAS concentration
**Zhang 2023**	24-28 GW	OGTT	IADPSG	1st Trimester	Mean	Tertiles	HPLC LC-MS/MS	NA	NA	Crude and adjusted OR (GDM)	By tertile & per log10-unit difference in PFAS concentration

^a^ Adjusted for subcohort only.

^b^ Conditioned on matching factor, maternal age.

To measure the association between PFAS concentration and glucose, the reports used associations between PFAS and one or more of the glucose measures: FG, 1hG, 2hG, OGTT (average of 1hG and 2hG), Impaired Glucose Tolerance (IGT), and GDM. Adjusted odds ratio (OR) (12 reports), crude OR (eight), adjusted regression coefficient (β) relating serum glucose to PFAS (nine), and crude β (three) were the main reported association measures. When a log exposure metric (comparator) was used, it was implemented heterogeneously with per one log-unit difference in PFAS concentration presented in nine studies, per log2 (doubling) difference in four, and per log10 difference in three. Eleven of the reports examined associations with exposure data categorized as either tertiles (six reports), quartiles (four), or dichotomized (one). The majority of these were in conjunction with exposure data examined as a continuous variable, but two reports displayed results with categorized PFAS concentration only, one as tertiles and one as quartiles of exposure.

We summarized the type of data in the original reports identified by our literature review according to the comparator and outcome ( Table 7). Results from reports that reported an effect measure for GDM (e.g., odds ratio) with exposure categorized into more than two categories were counted in the table as being arithmetic comparators because the results can be re-expressed that way. ^48^ For GDM, we found that 12 reports could be combined in a SWiM analysis for the two major PFAS. For reports presenting effect measures for glucose during pregnancy (same two PFAS), we found 11 results that could potentially be combined using SWiM. If results for GDM and glucose were combined in a SWiM, results for 17 could be included – assuming the participants in Wang *et al*. 2023a were a subset of those in Yu *et al*. 2021.

**Table 7.** Number of reports that could be included in a meta-analysis or SWiM by type of outcome and type of comparator.

Type of outcome	Type of comparator	N of reports *	
		PFOA	PFOS
Gestational Diabetes Mellitus	Arithmetic (ng/ml) †	8	8
	Log (ng/ml) ‡	9	9
	SWiM ^§^	12	12
Glucose during pregnancy	Arithmetic (ng/ml) †	3	3
	Log (ng/ml) ‡	8	8
	SWiM ^§^	11	11

* N of studies using total amount of PFOA or PFOS.

† Includes studies with categorical results only that have more than two categories of exposure because these can be re-expressed as ng/ml.

‡ Regardless of the log of base used. For example, studies reporting difference in outcome per doubling of exposure (base log 2) are counted, as well as those using natural log or base 10 log.

§ Number of unique studies that could be included in a synthesis without meta-analysis. For example, if a study reported one set of results using an arithmetic comparator and another set of results using a log comparator, that study would be counted only once towards the number in the SWiM.

## Discussion

This scoping review of the evidence and methods used in primary and secondary data on the relation of GDM with PFAS addressed the overall question of whether a new systematic review was needed. Because data from several new reports were available since previous systematic reviews were conducted and because SWiM ^33^ could make the best use of all available data, an updated systematic review using SWiM would be a valuable addition to the literature.

The variety of comparators used in the original reports poses challenges to the use of meta-analysis to summarize data. If the original reports include a categorical exposure scale, a meta-analysis using a high:low exposure category comparator has been presented as an option. ^24^ The weaknesses of this approach, however, merit discussion. For PFAS, the exposure contrast corresponding to the high:low comparison is likely to vary substantially across studies. If a dose-response relation is operative, the magnitude of the meta-analytic summary would reflect the distribution of exposure in the studies, and the validity of the resulting meta-analytic statistics associated with such a summary is questionable. ^49^ Furthermore, categorical results within-study can be re-expressed to an arithmetic effect measure, which solves the above-mentioned problem and has the further benefit of using, rather than a portion, all the available data. Another issue is that if arithmetic and logged exposure measures were interchangeable after some type of re-expression, that would facilitate meta-analysis. But such re-expression does not work reliably. ^50^ An interesting example of the underlying problem can be found in a recent report by Padula *et al*. (2023). ^51^ They analyzed individual-level data on birth weight and serum PFOA first using log (exposure) and again using arithmetic exposure. The association was inverse in both analyses, but it was statistically significant when log (exposure) was used but not when arithmetic exposure was used. The SWiM is the only widely-accepted non-narrative approach to synthesizing the data from evidence streams based on different comparators. ^33^ The three previous systematic reviews each had methodologic elements yielding questionable results. Specifically, a) Gao *et al*. ^30^ did not provide an overall summary of results from different evidence streams, b) Wang *et al*., ^31^ by using a high:low comparator, ignored part of the data and combined results in the face of varying exposure contrasts, and c) Yan *et al*., ^32^ combined results across comparators, rendering nebulous the statistics associated with the results.

Another potential improvement in a future systematic review of the GDM-PFAS association would be to integrate data from the GDM-PFAS and blood glucose-PFAS evidence streams. Among studies of blood glucose as the only outcome (no GDM analysis), four out of the five included participants with GDM. Because blood glucose is near to normally distributed, the odds ratio for GDM and the beta coefficient for blood glucose are, with re-expression, interchangeable metrics. ^52^ Thus, data from the glucose-PFAS reports could be used to address the overall question. Even without such re-expression, SWiM could be used to combine the evidence streams.

Results for five PFAS (PFOA, PFOS, PFHxS, PFNA, and PFDA) were reported in almost all the original data studies because these are the PFAS that have the highest median serum concentration in background-exposed populations. In the previous systematic reviews, Gao *et al*. (2021) reported results for all five ^30^; Wang *et al*. (2022) reported on four, ^31^ and Yan *et al*. (2022) reported on these five plus three additional PFAS. ^32^ PFAS with lower median concentrations could have been analyzed by the original authors with the comparator > LOD vs not, and such results have not, to date, been included in meta-analyses; however, they could be included in a SWiM.

When conducting a meta-analysis of odds ratios for GDM, an assumption is that the log of the odds ratio per comparator unit (beta coefficient) does not vary by the prevalence of GDM in the underlying populations. The prevalence of GDM in the underlying populations is a function of the diagnostic criteria, the prevalence of obesity, and other study or population characteristics. ^53^^–^^55^ Whether these characteristics modify the beta coefficient could be evaluated in a meta-regression; such regression was not done in the previous meta-analyses.

Time has passed since the date of the search for relevant original data reports. Our search for relevant systematic reviews, however, was much more recent, and it is this search that is most important for our conclusions to remain valid. The number of new reports available was not as important for our conclusions as the finding that the methods employed in previous systematic reviews could be substantially improved.

## Data Availability

All data underlying the results are available as part of the article and no additional source data are required. Open Science Framework: Gestational diabetes mellitus in relation to serum per- and polyfluoroalkyl substances: A scoping review to evaluate the need for a new systematic review. https://doi.org/10.17605/OSF.IO/8U3XF. This project contains the following extended data:
• Search Strategies.docx
