# Supplementary material for: Gestational diabetes mellitus in relation to serum per- and polyfluoroalkyl substances: A scoping review to evaluate the need for a new systematic review
Source: F1000Res. 2024 Oct 9;12:1595. Originally published 2023 Dec 14. [Version 2] doi: 10.12688/f1000research.144376.2 (PMC11474155; doi:10.12688/f1000research.144376.2)
Supplement: Peer review history

# Reviewer report: Reviewer response for version 2

**DOI:** 10.5256/f1000research.172869.r330434

**Published:** 2024-10-14

**Authors:** Brice Nouthé, Evrard Kepgang

**Affiliations:** 1 McGill University, McGill, Canada; 2 Epidemiology, University of Antwerp Research Group Grammar and Pragmatics, Antwerp, Flanders, Belgium

Great work done: Kudos!

 No further changes needed at this juncture.

Are the rationale for, and objectives of, the Systematic Review clearly stated?

Yes

Is the statistical analysis and its interpretation appropriate?

Not applicable

If this is a Living Systematic Review, is the ‘living’ method appropriate and is the search schedule clearly defined and justified? (‘Living Systematic Review’ or a variation of this term should be included in the title.)

Not applicable

Are sufficient details of the methods and analysis provided to allow replication by others?

Yes

Are the conclusions drawn adequately supported by the results presented in the review?

Yes

Reviewer Expertise:

Diabetes Mellitus

We confirm that we have read this submission and believe that we have an appropriate level of expertise to confirm that it is of an acceptable scientific standard.

# Reviewer report: Reviewer response for version 1

**DOI:** 10.5256/f1000research.158160.r324731

**Published:** 2024-09-28

**Authors:** Raju Kanukula

**Affiliations:** 1 Monash University, Victoria, Australia

This scoping review was conducted very well without any issues, however I have the following concerns.

 1) In the rationale, authors could have explained the causative pathway between PFAS and GDM. 

 2) What is the rationale behind excluding review articles on this topic, but included meta-analyses.

 Apart from these, I have no other concerns. 

Are the rationale for, and objectives of, the Systematic Review clearly stated?

Partly

Is the statistical analysis and its interpretation appropriate?

Not applicable

If this is a Living Systematic Review, is the ‘living’ method appropriate and is the search schedule clearly defined and justified? (‘Living Systematic Review’ or a variation of this term should be included in the title.)

No

Are sufficient details of the methods and analysis provided to allow replication by others?

Yes

Are the conclusions drawn adequately supported by the results presented in the review?

Yes

Reviewer Expertise:

Methodologist and pharmacist.

I confirm that I have read this submission and believe that I have an appropriate level of expertise to confirm that it is of an acceptable scientific standard.

# Response

**Published:** 2024-10-01

**Authors:** Farhad Shokraneh

**Comment**: 1) In the rationale, authors could have explained the causative pathway between PFAS and GDM.

**Response**: Thank you for reading our review and for your comment. This scoping review examined the studies that assess the ‘Association’ from the observational studies. Considering that confounding factors such as obesity—as mentioned in the introduction—can contribute to GDM, we are unable to discuss ‘Causation’ and have yet to see research suggesting such a mechanism.

**Comment**: What is the rationale behind excluding review articles on this topic, but included meta-analyses.

 Apart from these, I have no other concerns.

**Response**: We excluded the narrative review articles with no usable data or a list of included studies. We, however, studied the systematic reviews and meta-analyses to identify more relevant studies for a possible systematic review in the future, avoid duplicate work, and discuss the suitability of conducting meta-analysis in these reviews.

# Reviewer report: Reviewer response for version 1

**DOI:** 10.5256/f1000research.158160.r296883

**Published:** 2024-07-16

**Authors:** Brice Nouthé, Evrard Kepgang

**Affiliations:** 1 McGill University, McGill, Canada; 2 Epidemiology, University of Antwerp Research Group Grammar and Pragmatics, Antwerp, Flanders, Belgium

The authors aimed to evaluate the evidence on a potential relationship between Gestational Diabetes Mellitus (GDM) and serum per-&polyfluoroalkyl substances (PFAS). This sponsored effort (by 3M) to evaluate the risk, caused by this group of synthetic carbon-fluorine compounds ensuring slow degradation, is timely because of the important issues evolving around 

- the demonstrated relationship of PFAS, known to have an extensive human exposure, and diabetes mellitus
- the different concerns about GDM across continents despite known lifelong consequences of GDM
- the potential conflictual results of previous meta-analysis.

Suggestion1: The authors used studies publicly available & sought to present the need for an updated systematic review before even looking at new source of information. We believe they do not need to expand the Introduction section because it could just be around the motivation of the study.

 Suggestion 2: In the Methods' section, the justification to use PECO & description the different strategies to retrieve the data could easily be included in a Supplemental Material . Also, the procedure to solve disagreement does not need to be repeated.

 Suggestion 3: The flow in the Results' section about the in-depth assessment of the 3 previous meta-analyses could be less of a narrative part but rather separated around he potential pitfalls like

- the use of meta-analyses with different comparators ( but without meta-regression)
- The combination of results form different evidence streams without appropriate summary
- The variability of  exposure contrasts also rendering the use of a high-low exposure category comparator much questionable.

Are the rationale for, and objectives of, the Systematic Review clearly stated?

Yes

Is the statistical analysis and its interpretation appropriate?

Not applicable

If this is a Living Systematic Review, is the ‘living’ method appropriate and is the search schedule clearly defined and justified? (‘Living Systematic Review’ or a variation of this term should be included in the title.)

Not applicable

Are sufficient details of the methods and analysis provided to allow replication by others?

Yes

Are the conclusions drawn adequately supported by the results presented in the review?

Yes

Reviewer Expertise:

Diabetes Mellitus

We confirm that we have read this submission and believe that we have an appropriate level of expertise to confirm that it is of an acceptable scientific standard, however we have significant reservations, as outlined above.

# Response

**Published:** 2024-10-01

**Authors:** Farhad Shokraneh

**Suggestion 1**: The authors used studies publicly available & sought to present the need for an updated systematic review before even looking at new source of information. We believe they do not need to expand the Introduction section because it could just be around the motivation of the study.

**Response**: Thank you for spending time reviewing this scoping review and for your suggestion not to expand the introduction.

**Suggestion 2**: In the Methods' section, the justification to use PECO & description the different strategies to retrieve the data could easily be included in a Supplemental Material.

**Response**: Thank you. We followed your recommendations and F1000Research’s guidelines and shared the search strategies on the Open Science Framework (OSF).

**Comment**: The procedure to solve disagreement does not need to be repeated.

**Response**: We agree with your decision and have revised the manuscript to reflect it.

**Suggestion 3**: The flow in the Results section about the in-depth assessment of the 3 previous meta-analyses could be less of a narrative part but rather separated around the potential pitfalls like

- The use of meta-analyses with different comparators (but without meta-regression)
- The combination of results from different evidence streams without appropriate summary
- The variability of exposure contrasts also rendering the use of a high-low exposure category comparator much questionable.

**Response**: Thank you for your suggestion, based on which we changed the first section of results as follows:

 “In one systematic review (Yan et al., 2022), the use of meta-analysis to combine results across comparators rendered the meaning of the summary OR obscure. In another systematic review (Gao et al., 2021), results from different evidence streams were not combined using an appropriate summary. In the third systematic review (Wang et al., 2022), the focus on only high:low exposure category comparators meant: a) informative data from each included study were ignored, b) the variability of the exposure contrasts made use of this comparator questionable, and c) reports using only models with exposure as a continuous variable were ignored.”
